# Editorial: On the Nature and Scope of Habits and Model-Free Control

**DOI:** 10.3389/fpsyg.2021.760841

**Published:** 2021-10-21

**Authors:** David E. Melnikoff, John A. Bargh, Wendy Wood

**Affiliations:** ^1^Department of Psychology, Northeastern University, Boston, MA, United States; ^2^Department of Psychology, Yale University, New Haven, CT, United States; ^3^Department of Psychology, University of Southern California, Los Angeles, CA, United States

**Keywords:** habit, automaticity, motivation, reinforcement learning, model-free, model-based

There is wanting to do something, and there is actually doing it. About 70% of smokers say they want to quit, yet fewer than one in ten succeed each year (Centers for Disease Control Prevention CDC, 2017). Nearly 50% of Americans are trying to lose weight (McCarthy, 2021)—an all-time high—yet the body mass index of the average American is going up, not down (Warren et al., 2020). Quitting social media was among the top new year's resolutions of 2017 (Alexander, 2018), yet the rate of social media use hasn't budged (Pew Research Center, 2021).

What is stopping so many of us from doing what we want to do? Much of the blame gets placed on our habits. When maladaptive behaviors become habitual, it can be incredibly difficult to maintain a healthy, happy, and productive lifestyle. It is no surprise, then, that research on habit connects across disciplines, including neuroscience and artificial intelligence, health psychology and animal learning, social psychology and cognitive psychology, philosophy and anthropology. Habits have been analyzed at multiple levels (Marr, 1982). At the functional level, psychologists have explored the inputs to the habit-formation process (e.g., reward, punishment, behavioral repetition, context cues), and the basic logic of how these inputs interact to produce habits and control behavior (James, 1890; Hull, 1943; Dickinson, 1985; Wood and Neal, 2007; Gardner, 2015; Wood and Rünger, 2016; de Wit, 2017; Knowlton and Diedrichsen, 2018). At the algorithmic level, work in artificial intelligence has generated sophisticated accounts of the specific computations underlying habit formation and habitual control (Botvinick and Plaut, 2004; Daw et al., 2005; Doll et al., 2012; Dolan and Dayan, 2013; Miller et al., 2019). At the implementational level, neuroscientists have worked to ground algorithmic and computational insights in underlying neural substrates (Packard et al., 1989; Knowlton et al., 1996; Patterson and Knowlton, 2018). This cross-disciplinary, multi-level approach has produced a wealth of insight and is a generative source of concepts, frameworks, and theories.

The classic definition of habit in psychology is a mental association between a stimulus and a response that develops through repeated instrumental learning (Amodio, 2019). Along with articles that further establish and extend this standard conception, this Research Topic, On the Nature and Scope of Habits and Model-Free Control, includes alternatives to current habit theorizing and accepted definitions, including action-value associations, episodic memory traces, and action-outcome associations. As researchers continue to push the boundaries of habit theory, we encourage the reader to think of this Research Topic as a roadmap for navigating this rapidly growing conceptual space. In what follows, we orient the reader by summarizing the primary school of thought in habit research, along with the evidence and arguments that contributors advanced for it and, at times, for their favored alternative.

## Habits as Stimulus-Response Associations

Habitual responses are most often described as emerging from stimulus-response associations in memory (Thorndike, 1905; Skinner, 1938; Hull, 1943; Wood and Rünger, 2016; Knowlton and Diedrichsen, 2018; Verplanken and Orbell, 2021). These associations are thought to emerge from behavioral repetition, and the speed with which they emerge is thought to be modulated in part by reward, such that stimulus-response pairings that predict rewarding outcomes are privileged. Once a stimulus-response association is sufficiently strong, the stimulus itself becomes capable of activating the action automatically when it is re-encountered (a process called *direct cuing*; Wood and Rünger, 2016).

This conceptualization of habit explicitly guided the work of six of our contributors and provided a broader context for the remaining contributions. Patterson et al. explored how early life stress relates to “stimulus–response habits,” defined as “instrumental behaviors that… have come to be automatically elicited by stimuli in whose presence the behavior has been repeatedly performed, without regard to instrumental outcomes.” Also using this classic treatment of habit, Ceceli et al. investigated the relationship between “existing, well-learned S-R associations” and attention-deficit/hyperactivity disorder (ADHD). Verplanken and Sui explored the causes and consequences of deriving a sense of identity from one's stimulus-response habits. Finally, McCloskey and Johnson explored how the complexity of an action moderates the habit formation process, described as follows: “When a behavior is performed regularly in a stable context, the individual is more likely to encounter consistent cues that can form the basis for a context-behavior association. As frequency of this behavior increases, so too can the strength of the context-behavior association.”

Within this stimulus-response framework, each article provides important insights. Patterson et al. found a positive effect of early life stress on the strength of avoidance habits (i.e., habits formed over the course of avoiding negative outcomes). As the authors note, this finding points to an important role of habit in mediating the relationship between early life stress and negative health outcomes. A range of negative health behaviors, such as overeating, substance abuse, and risky sexual behavior, might be performed initially in order to avoid feelings of distress and then with repetition transition into avoidance habits. This tendency to act on habit represents a behavioral vulnerability that could account for the poor health outcomes associated with early life stress. Further applying a habit analysis to attention deficit/hyperactivity disorder (ADHD), Ceceli et al. found that ADHD had, at most, a limited relationship with habitual control. This is surprising in light of animal behavior and neuroscience research linking habit to ADHD symptomology and, as these authors speculated, the limited effects could be due to insensitivity of their particular experimental task.

Continuing the theme of linking habit to health, Verplanken and Sui found that self-esteem—a powerful predictor of mental and physical health—was greater to the extent that people felt their true self was reflected in value-relevant habits (e.g., buying ecological products). That is, well-being benefits when people repeatedly act in ways that support their self-concept. On the one hand, self-expression could be a kind of reward that promotes repetition and habit formation of value-relevant behaviors. On the other, values may be inferred in part from observing repeated actions, so that habit formation drives self-identity. Both causal routes are plausible, and they help to explain how habit is a foundational element in favorable self-views and well-being (Heintzelman and King, 2019).

Given their influence on health outcomes, habits in noisy real-world settings are especially important for psychologists to predict and measure. This challenge was taken up by McCloskey and Johnson (2019), who estimated the relative influence of reward, behavioral repetition, context stability, and behavioral complexity on real-world habit formation as reflected in judgments of perceived automaticity. As expected, across a number of everyday behaviors, people performed actions more automatically when those actions were more rewarding, performed in stable contexts, and repeated more often. Reward and context stability were especially important for complex behaviors; as behavioral complexity increased, so did the effects of reward and context stability on habit strength.

In addition to the daily behaviors studied by McCloskey and Johnson (2019), simple behaviors, such as a phone-app-based motor sequence, also become more automatic with daily practice (Banca et al.). This work importantly demonstrated that practice heightened automaticity as reflected in (a) reduced sequence completion times, (b) progressively less reliance on learning cues, (c) decreased variability in finger movements, and (d) autonomy from the goal as assessed by extinction (removal of explicit reward feedback). These four indicators of automaticity illustrate how habit formation can be tracked through a rich set of behavior data, thereby extending existing research on habit to include often-overlooked features of automaticity, especially performance variability. The compatible effects across all four indicators provide especially strong support for the stimulus-response view of habit learning and suggest that mobile phone apps could play an important role in advancing the study of habit.

Habitual responding is, of course, only one form of action control, and some of the most valuable contributions in this Special Topic outline how stimulus-response habits integrate with other response systems, especially goal-directed control. Although competition between habit and goal pursuit is often baked into the design of habit research, as habit performance is most convincingly demonstrated when in conflict with desired outcomes, Balleine and Dezfouli (2019) argue instead for a more collaborative relation. They propose that habitual actions are activated by associated cues that directly trigger a motor response as well as an action representation, which in turn retrieves the action outcome and its evaluation. In this model, responses are a function of the evaluative system's feedback signal in combination with the forward excitation from habit memory. These processes align in a hierarchical fashion in which habits arise from the aggregation of individual actions into unitary, “chunked” representations (Dezfouli and Balleine, 2012, 2013; Dezfouli et al., 2014). A regular tea drinker, for instance, may combine the actions *put teabag in teapot, pour hot water into teapot*, and *pour tea into cup* into the single, unified action representation, *make tea* (Cooper and Shallice, 2000; Botvinick and Plaut, 2004). Once the chunk is selected for performance, the elements within the chunk run off on auto-pilot, such that the execution of one action in the sequence (e.g., put teabag in teapot) automatically triggers the next action (e.g., pour hot water into teapot). In this setting, the actions within the chunk are habitual in the sense that they are triggered automatically by a stimulus (i.e., the preceding action and the current state of the environment—pot with bag but no water). At the same time, the actions within the chunk can be thought of as goal-driven in the limited sense that the chain of events leading up to their triggering was initiated by a goal to act. One important contribution of Balleine and Dezfouli's research is the use of reaction times to isolate the speedy activation of habitual responses as opposed to the slower valuation of goal-directed responding—a feature of habit that has broad implications for habit performance (see Hardwick et al., 2019; Wood et al., 2021).

The question of how habits integrate with goal-directed actions is further addressed by Eder and Dignath who challenge the common answer that a cognitive controller intervenes against a habit controller (i.e., default interventionist model). Instead, they draw on research that links habitual responses with Pavlovian instrumental transfer to argue for a single cognitive system oriented by the expected value of control. In this view, people respond automatically out of habit to the extent that the expected value of the habitual response outweighs the intrinsic cost of exerting effortful control to select an alternative. It may be, then, that commonly ascribed features of habit, such as insensitivity to changes in outcome values, reflect not intrinsic habit features but instead the efficiency-driven cognitive system that controls action tendencies.

## Habits as Action-Value Associations

Elsewhere in this Special Topic, researchers emphasize repeated learning of action-value associations (Hackel et al., 2019; Morris and Cushman, 2019). This is the “model-free” account of habit, which derives from the field of computational reinforcement learning (Sutton and Barto, 1998; Daw et al., 2005; Dolan and Dayan, 2013). In this account, cognitive systems use rewards and punishments to compute the long-run value of performing a given action in a given context. Armed with these simple action-value associations, agents select actions on the basis of expected long-run value rather than conduciveness to the agent's current needs, desires, and knowledge of the causal structure of the environment (a process called *motivated cuing*; Wood and Neal, 2007).

Model-free RL and classic habit theory align in various ways. In both accounts, people respond on the basis of past rewards rather than current goals and estimations of response outcomes. This basic feature of habit was initially demonstrated in *reinforcer devaluation* studies of animal learning, which showed that animals extensively trained on a task persist despite changes in rewarding outcomes (extinction, devaluation) or the response-outcome contingency (e.g., Dickinson, 1985). Furthermore, both habit and model-free learning are the result of prior active responding and thus differ from more passive associative learning, such as classical conditioning.

Yet, the specific logic of model-free learning and habit differ in several ways. For model-free learners, for example, choice cues acquire cached representations of past reward values, whereas habitual learners respond to simple cue-response associations. This difference in emphasis is associated with different research paradigms, with stimulus-response researchers studying a variety of instrumental learning tasks with stable reward structures (see Patterson and Knowlton, 2018), and model-free learning largely tested with a specific two-stage decision task in which the rewarded response varies across trials. Despite these differences, Hackel et al. argue that model-free learning has many of the features of habits and suggest that both processes contribute to behaviors commonly considered habitual. Similarly, Morris and Cushman (2019) suggest that model-free responses map onto intuitive notions of habitual behaviors.

Hackel et al. adapted the two-stage task from model-free learning to explore the role of habit in the formation of social preferences. Their innovative research is some of the first demonstrating that habits contribute to the persistence of interaction choices despite current goals. The more participants were rewarded for interacting with someone, the more they liked that interaction partner, and the more often they interacted with that partner again, independent of their current knowledge about the partner's value. In other words, attitudes and social choices reflected not only goal-based decisions about how rewarding a social partner was expected to be in the future but also habitual choices of how rewarding that partner had been in the past.

Morris and Cushman (2019) adapted the two-stage task to evaluate the mental representations behind discrete choices. Specifically, they tested whether habitual choices reflect model-free reinforcement learning or the goal-based selection of multiple actions that have been combined into a single representation (see Balleine and Dezfouli, 2019). Their results suggests that both mechanisms—model-free reinforcement learning and model-based selection of “chunked” action sequences—contribute to performance. Morris and Cushman (2019) provide an especially thoughtful analysis of how to interpret the response representations in this classic task, and conclude that “the puzzle of habits will not be solved by one model; ‘habits’ likely comprise multiple decision strategies, including both model-free RL and action sequences.” In this way, they encourage researchers to consider the variety of response representations that could account for persistence within the two-stage task structure.

## Alternative Treatments of Habits

People learn many different types of associations in interacting with the environment, and some of the articles in the Special Topic extend habit to include these other forms of associative learning. These analyses are interesting to compare with the more standard treatment of habit as learning of context-response associations.

In an extension of existing habit theorizing, Giesen et al. proposed that habits can form according to Thorndike's law of recency, along with the more standard laws of effect (reward learning) and exercise (repetition). That is, performing a behavior in a specific context increases the likelihood of performing that behavior when the situation is encountered again in the near future, even in the absence of reward and repeated responding. This analysis thus builds on episodic memory models of automatic responding. In support, their novel color-categorization paradigm revealed independent contributions of stimulus-response learning and episodic memory traces to responding, and furthermore demonstrated that recency of presentation was a better predictor of responses in this task than past repetition. However, recency is unlikely to explain repetition in standard habit formation paradigms, which typically hold recency constant while manipulating the amount of practice in weak and strong habit conditions.

Several papers challenge classic theorizing by proposing to incorporate habit processes within a single action control system that is structured by the pursuit of goals. For example, Sun et al. explored whether people spontaneously learn action-outcome associations in two distinct phases of behavioral processing in instrumental learning tasks: action selection and action initiation. This involved establishing action-outcome associations, and then testing whether the outcomes, when presented as primes, facilitate the actions with which they had been associated. Given that outcomes facilitated their associated actions in a forced-choice paradigm but not a free-choice paradigm, action-outcome links were assumed to support action selection over initiation. De Houwer further questioned the stimulus-response definition of habit and argued instead for a less specific definition that could include a variety of mechanisms (e.g., episodic memory traces, predictive models). By defining habits without reference to stimulus-driven responding, De Houwer challenges much of the existing research on habit to advocate for a single model of action control in which goals intervene between environments and behaviors.

Also incorporating habit responding within a single system, goal-driven model, Hommel argued that all actions are chosen on the basis of whether they meet the selection criteria defined by the actor's current goals. The broad goal to act quickly and easily leads to a preference for well-practiced actions, given that responding becomes faster and easier with practice. According to Hommel, this basic principle, in the absence of a second system dedicated to habitual control, explains why behavioral repetition promotes habit formation. O'Reilly et al's. computational model similarly blurs distinctions between habitual and goal-directed action. Specifically, they argue that all habits start with a goal-based decision about whether to allow habit automaticity to proceed. An initial component—the *proposer*—takes top-down and bottom-up information as input, and outputs a single representation as a candidate for action. This proposal is sent to the *predictor*, which anticipates the specific outcome that the proposed action would produce and how valuable that outcome would be given the agent's current goals. The proposed action, along with its predicted outcomes, are sent to the *actor*, which either accepts or rejects the proposal. If the proposal is accepted, the action is performed and, if not, the process starts anew. Under this model, when speed is at a premium, the actor will favor habitual actions by selecting the first one proposed. Similar to Hommel, habits in this model are goal-dependent in the sense that the goal for fast, efficient responding must be in place for habits to emerge. It remains to be seen in future research how well these single-model systems fare in accounting for habitual patterns of responding.

## A Roadmap for Habit Research

Habits are most often described as reflecting stimulus-response associations that compete against goal-directed processing for control over behavior. Yet modern habit theory frequently supplements this view, and at times challenges it, with alternative accounts that connect habits to different types of representations and processes. The situation raises the intriguing possibility of a greater degree of complexity than merely a single type of representation and a single type of process. This point is nicely illustrated by Morris and Cushman, who devised a paradigm that simultaneously measures collaborative processing, as envisioned by Balleine and Dezfouli, and competitive processing, as envisioned by standard approaches to habit. Their results suggest that both types of processes contribute to habitual behavior, providing an important clue in the search for the true nature of habit. Nature tends toward complexity, and results like those of Morris and Cushman suggest that habits are no exception.

In our view, however, the complexity is bounded at both ends. While a single representation and process is likely to be an oversimplification, it is also not the case that all possible combinations of features are equally likely. The popular System 1 and System 2 model of human decision-making is a case in point. Even its most prominent supporters acknowledge it is really just a convenient fiction (Kahneman, 2011, p. 54) and there are not, in reality, two and only two types of thought. Evans and Stanovich's 2013 defense of dual process models recognized a heterogeneous set of System 1 processes, including innate modules and experiential associations learned to the point of automaticity. Thus, mental processes have many different qualities, and each type of process can vary along a number of somewhat independent dimensions (Melnikoff and Bargh, 2018). This results in multiple systems (Amodio, 2019), not just two—but it does not follow from the notion of multiple systems that human psychology proceeds through a random combination of different independent features. For example, some features of automaticity are naturally correlated and tend to occur together (Evans and Stanovich, 2013), such as awareness and control, because it is more difficult (but not impossible) to control a process one is not aware is operating. Just as we should not let our human fondness for simplicity cloud our scientific vision as to the realities of habit formation and operation, we also should not conclude that it is some random process, and that all models are somehow equally valid. Of course, the most important reason for this bounded complexity view is the consistent evidence on habit learning and performance from now decades of research.

As we noted in starting this article, research evidence on habit strongly suggests that there is a collection of features of automaticity that cluster together and guide future behavior when people have repeated an activity in the same way. Habits are compatible with a multiple systems analysis. They suggest a certain kind of complexity in processing, one that acknowledges goal-driven action (which has certain distinguishing features, long noted, such as continued activation and operation until the goal is met) along with classical conditioning influences and more reflexive, S-R types of responding. Thus, habits were the single system that Skinner (1938) and Hull (1943) signed on to and at the same time are now part of the multiple system neuroscience-based analyses of Squire and Zola-Morgan (1991) and Amodio (2019). This research evidence also points to a mental representation for habit that links context cues with responses and is relatively insensitive to goals. We acknowledge that not all of the papers in this special issue recognize or endorse this position, perhaps because of the different emphases, experimental paradigms, and target domains of the various subfields of psychology, but it is the view consistent with the empirical body of knowledge in the field of habit research.

So yes, there is something “there,” there. There is a coherent construct of *habit* that at the same time involves multiple systems. Not just one, but neither an unconstrained number of combinations of important information processing features. The articles in this special issue exemplify the terrific progress that psychology and its related fields have made in bringing the construct of habit into ever sharper focus. Together they form a sturdy platform on which to build further advances in our knowledge of this great flywheel of human society and everyday life.

## Author Contributions

All authors listed have made a substantial, direct and intellectual contribution to the work, and approved it for publication.

## Conflict of Interest

The authors declare that the research was conducted in the absence of any commercial or financial relationships that could be construed as a potential conflict of interest.

## Publisher's Note

All claims expressed in this article are solely those of the authors and do not necessarily represent those of their affiliated organizations, or those of the publisher, the editors and the reviewers. Any product that may be evaluated in this article, or claim that may be made by its manufacturer, is not guaranteed or endorsed by the publisher.
